# Design, Synthesis and Antiproliferative Activity of Novel 2-Substituted-4-amino-6-halogenquinolines

**DOI:** 10.3390/molecules17055870

**Published:** 2012-05-16

**Authors:** Nan Jiang, Xin Zhai, Ting Li, Difa Liu, Tingting Zhang, Bin Wang, Ping Gong

**Affiliations:** Key Lab of New Drugs Design and Discovery of Liaoning Province, School of Pharmaceutical Engineering, Shenyang Pharmaceutical University, 103 Wenhua Road, Shenhe District, Shenyang 110016, China

**Keywords:** 2-substituted-4-amino-6-halogenquinolines, design, synthesis, antiproliferative activity

## Abstract

Two series of novel 2-substituted-4-amino-6-halogenquinolines **8a**–**l** and **13a**–**h** were designed, synthesized and evaluated for their antiproliferative activity against H-460, HT-29, HepG2 and SGC-7901 cancer cell lines *in vitro*. The pharmacological results indicated that most compounds with 2-arylvinyl substituents exhibited good to excellent antiproliferative activity. Among them, compound **8e** was a considered promising lead for further structural modifications with IC_50_ values of 0.03 μM, 0.55 μM, 0.33 μM and 1.24 μM, which was 2.5- to 186-fold more active than gefitinib and compound **1**.

## 1. Introduction

**Figure 1:**
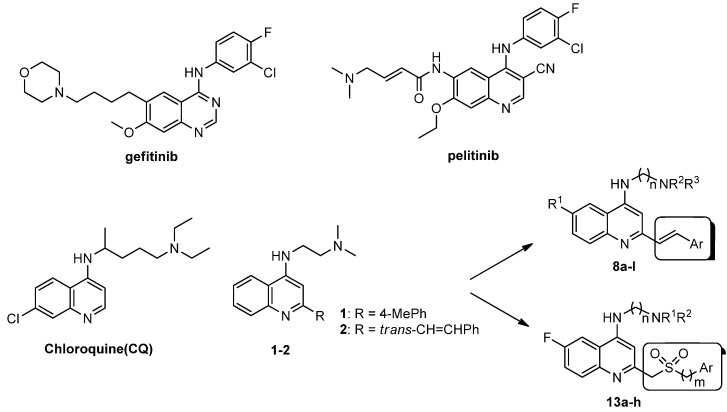
Structures of gefitinib, pelitinib, CQ, CQ derivatives and target compounds **8a-l**, **13a-h**.

Since a series of targeted antitumor agents such as gefitinib and pelitinib were approved for cancer therapy, the 4-aminoquinazoline and 4-aminoquinoline skeletons are considered to be promising nucleus for antitumor drug development [1]. As a result, a great number of novel 4-aminoquinazoline and 4-aminoquinoline derivatives have been developed in succession (Figure 1). Chloroquine (CQ), the worldwide used immunostimulatory agent with 4-aminoquinoline skeleton, has recently aroused increasing attention owing to its surprising antiproliferative potency on different cancer cells [2,3,4,5]. Similarly, Strekowski *et al.* developed a short series of 2-alkyl-4-aminoquinoline CQ derivatives (e.g., **1** and **2**) as immunostimulatory agents [6,7]. Among them, compound **1** with a 2-aryl group was also reported for its excellent antiangiogenesis activity in the chick embryo chorioallantoic membrane (CAM) assay [8], which brought us new hope in the study of antitumor agents. Under the inspiration of CQ and compound **1**, we conjectured that analogue **2** with a 2-styryl group should possess promising antiproliferative effects as well.

Prompted by this conjecture, we have designed two series of novel 2-substituted-4-amino-6-halogenquinolines. In our efforts to provide derivatives endowed with improved electron affinity and better biological interactions, substituents of various arylvinyl and alkylamino functional groups were introduced into the C-2 and C-4 positions, respectively, to generate compounds **8a–l**, which were prepared via an optimized Knoevenagel reaction condition. Meanwhile, the introduction of halogen at the C-6 position might adjust lipophilicity. In order to examine the effect of the pattern of the spacer between nucleus and aryl moiety on their activity, target compounds **13a–h** were synthesized through a methylsulfone or dimethylsulfone linkage. All target compounds were evaluated for their antiproliferative activity *in vitro* against four typical cancer cell lines (H-460, HT-29, HepG2 and SGC-7901 cell lines) and a preliminary SAR study of these compounds is discussed.

## 2. Results and Discussion

### 2.1. Chemistry

**Scheme 1:**
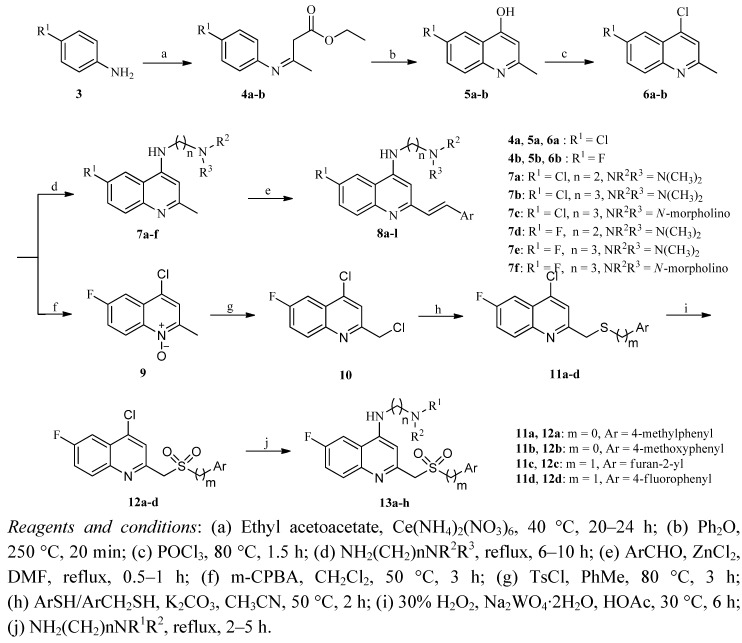
Synthesis of target compounds.

The synthetic routes of target compounds **8a–l** and **13a–h** are illustrated in Scheme 1. The commercially available ethyl acetoacetate was condensed with 4-substituted anilines in the presence of ammonium ceric nitrate at 40 °C leading to the formation of **4a–b** [9]. Treatment of **4a–b** in diphenyl ether at 250 °C for 20 min yielded compounds **5a–b**, which were then chlorinated with phosphoryl chloride to afford 4-chloro-2-methylquinolines **6a–b** in 97–98% yields [10,11]. Subsequent alkylation of **6a–b** with an excess of the corresponding aliphatic amines at reflux gave rise to the key intermediates **7a–f** [12].

According to the literature, 2-arylvinylquinolines can be prepared via Knoevenagel reaction of 2-methylquinolines and aryl aldehydes, using acetic acid as a solvent at 100 °C for 24 h [13], or with fused zinc chloride as catalyst for 2 h [14,15]. However, both methods failed due to the number of side reactions or the black viscous state of the reaction systems. After repeated experiments, an efficient modified condition for preparing 2-arylvinylquinolines **8a–l** in 74–92% yields, using zinc chloride as catalyst in reﬂuxing *N*,*N*-dimethylformamide (DMF) for 0.5–1 h, was successfully employed. 

It’s worth mentioning that this optimized method for the preparation of 2-arylvinylquinolines predominantly generates the (*E*)-isomer from simple building blocks. Coupling constants (*J* = 15–17 Hz) from the proton nuclear magnetic resonance (^1^H-NMR) spectra of target compounds clearly indicated that derivatives **8a–l** were both geometrically pure and were exclusively *trans* (*E*) isomers.

The oxidation reaction of 4-chloro-6-fluoro-2-methylquinoline (**6b**) with 3-chloroperbenzoic acid provided compound **9** [16], which was chlorinated with benzenesulfonyl chloride to afford 4-chloro-2-(chloromethyl)-6-fluoroquinoline (**10**) [17]. The reaction of **10** with substituted thiophenols or benzylthiols was carried out in the presence of potassium carbonate at 50 °C to give compounds **11a–d**, which were converted into compounds **12a–d** after treatment with 30% hydrogen peroxide and sodium tungstate in acetic acid [18]. Finally, target compounds **13a–h** were obtained according to the same method as described for compounds **7a–f**.

### 2.2. Biological Results and Discussion

The antiproliferative activity of two series of target compounds **8a–l** and **13a–h** was evaluated with non-small-cell lung cancer cell line (H-460), human colon cancer cell line (HT-29), human liver cancer cell line (HepG2) and stomach cancer cell line (SGC-7901) by the MTT assay *in vitro*, with compound **1** and gefitinib as the positive controls. The results expressed as IC_50_ values are summarized in Table 1 and Table 2.

**Table 1:** The substituents and IC_50_ values of **8a–l** against H-460, HT-29, HepG2 and SGC-7901 cells *in vitro*.

Compd.	R^1^	n	NR^2^R^3^	Ar	IC_50_ (μmol/L)			
					H-460	HT-29	HepG2	SGC-7901
**8a**	Cl	3	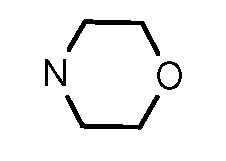	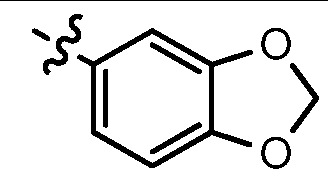	1.57	2.04	2.89	3.34
**8b**	Cl	3	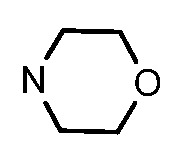	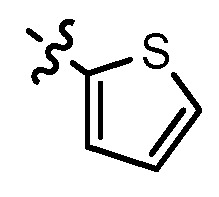	2.10	2.36	1.76	4.21
**8c**	Cl	2	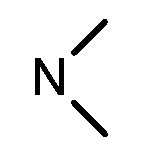	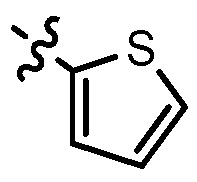	0.46	0.79	0.93	1.56
**8d**	Cl	3	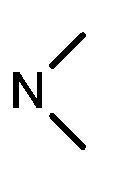	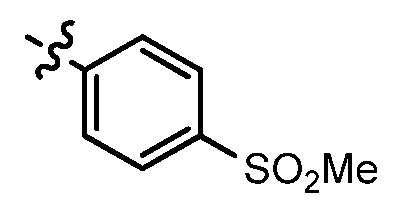	1.58	2.35	3.03	3.30
**8e**	Cl	2	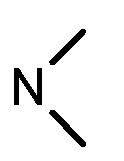	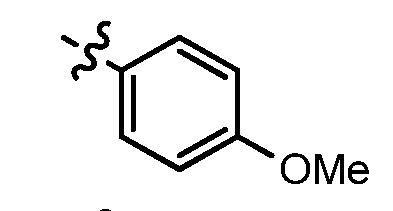	0.03	0.55	0.33	1.24
**8f**	Cl	3	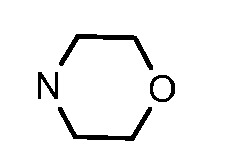	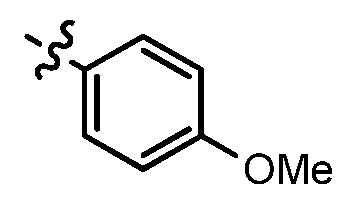	1.20	1.74	1.86	1.55
**8g**	F	2	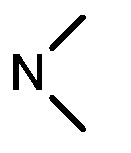	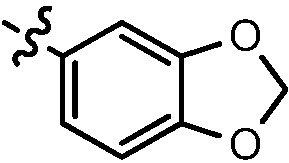	0.51	1.03	0.66	1.33
**8h**	F	3	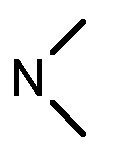	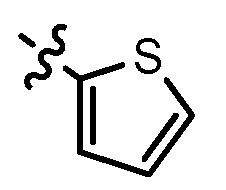	0.49	0.96	0.77	1.98
**8i**	F	2	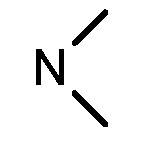	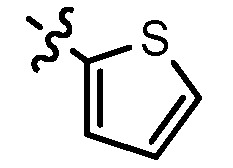	0.91	1.84	1.56	1.72
**8j**	F	3	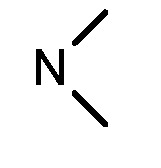	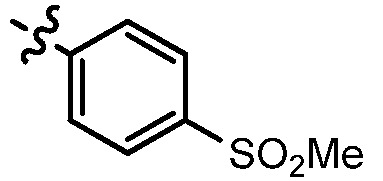	1.97	1.68	4.88	5.60
**8k**	F	2	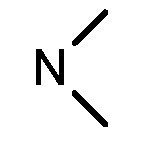	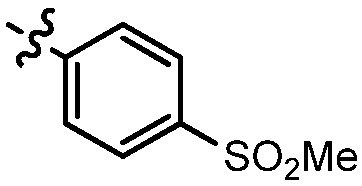	3.82	5.27	4.51	11.84
**8l**	F	3	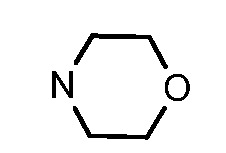	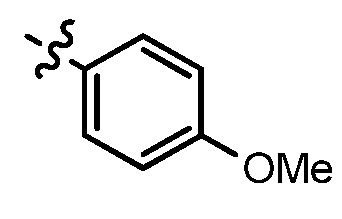	2.32	2.46	1.78	4.74
**1**					3.52	1.35	2.06	4.92
gefitinib					5.59	3.36	6.42	10.26

As shown in Table 1, all compounds except for **8k** exhibited good to excellent antiproliferative activity, with IC_50_ values ranging from 0.03–4.74 μM, which were 1.1- to 186-fold better than the positive controls. Among them, compound **8e** bearing a 4-methoxystyryl group at the C-2 position and a 3-(dimethylamino)-1-propylamino substituent at the C-4 position possessed the best antiproliferative potency (IC_50_ values of 0.03 μM, 0.55 μM, 0.33 μM and 1.24 μM) and emerged as a lead for further research on quinoline analogues. Furthermore, almost all the compounds were more potent against H-460 cells than against other three cells, reﬂecting good selectivity for lung cancer.

In general, the substituents on the side chain at the C-4 position had a major influence on pharmacological activity, and variations of the terminal substituents on the side chain would change the activity dramatically. Generally, a dimethylamino group made a good contribution to the antiproliferative potency, while introduction of a morpholinyl group resulted in a certain decrease in activity (**8a** *vs*. **8g**, **8b** *vs*. **8h**, **8e** *vs*. **8f**), which might be due to its steric hindrance. 

**Table 2:** The substituents and IC_50_ values of **13a–h** against H-460, HT-29 cells *in vitro*.

Compd.	m	n	NR^1^R^2^	Ar	IC_50_ (μmol/L)		Compd.	m	n	NR^1^R^2^	Ar	IC_50_ (μmol/L)	
					H-460	HT-29						H-460	HT-29
**13a**	0	2	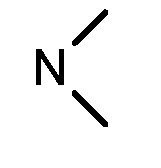	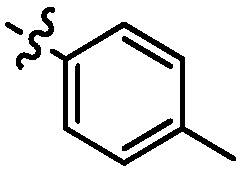	47.3	69.7	**13f**	1	2	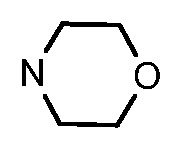	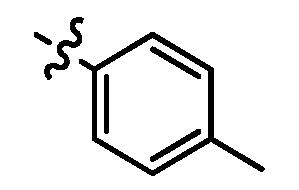	22.1	14.3
**13b**	0	3	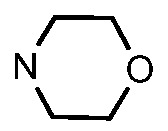	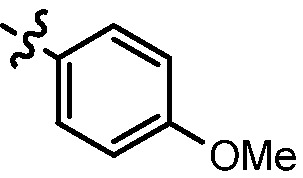	NA ^a^	185.2	**13g**	1	3	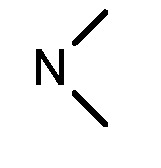	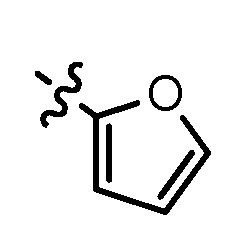	9.0	4.7
**13c**	0	3	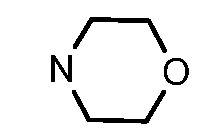	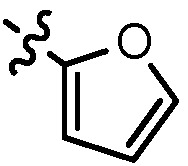	160.3	NA ^a^	**13h**	1	3	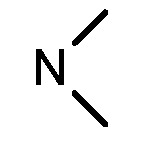	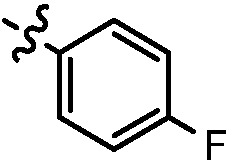	156.3	NA ^a^
**13d**	1	2	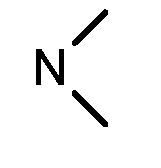	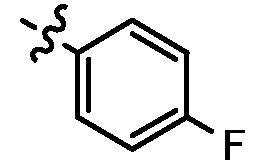	21.97	53.6	**1**					3.52	1.35
**13e**	1	3	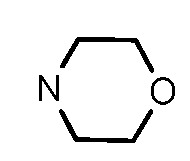	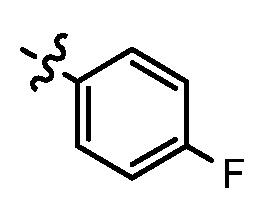	146.9	NA ^a^	gefitinib					5.59	3.36

^a^ NA: Compounds having IC_50_ value > 200 μM.

As can be seen from the comparison of Table 1 and Table 2, all compounds with 2-arylvinyl substituents were more active than those with 2-aryl, 2-(arylsulfonyl)methyl and 2-(benzylsulfonyl)methyl substituents. The results suggested that the introduction of ethylene linkages between nucleus and aryl moiety based on the structure of compound **1** was essential for improving activity, and a methylsulfone or dimethylsulfone linkage was not favorable in this region. Further analysis of 2-arylvinyl groups revealed that compounds substituted with electron donating groups were superior to that with strong electrophilic groups, and the greatest enhancement of activity occurred with the 4-methoxyphenyl substituent. A case in point is that 4-methoxysubstiuted compound **8e** was the most active compound, whereas **8k** bearing 4-methylsulfonyl substituent showed a 2.5- to 4-fold decrease in potency against HT-29, HepG2 and SGC-7901 cell lines compared to the positive control **1**.

## 3. Experimental

### 3.1. Chemistry

Melting points were obtained on a Büchi Melting Point B-540 apparatus (Büchi Labortechnik, Flawil, Switzerland) and were uncorrected. Mass spectra (MS) were taken in ESI mode on Agilent 1100 LC-MS (Agilent, Palo Alto, CA, USA). Proton (^1^H) nuclear magnetic resonance spectra were recorded using Bruker ARX-300 and Bruker ARX-600 spectrometers (Bruker Bioscience, Billerica, MA, USA) with TMS as an internal standard. Unless otherwise noted, all common reagents and solvents were used as obtained from commercial suppliers without further puriﬁcation.

### 3.2. General Procedure for the Preparation of Ethyl-(3Z)-3-(4-substitutedphenylamino)but-2-enoates ***4a–b***

Ammonium ceric nitrate (5 mmol) was added to a stirred solution of 4-substituted aniline (0.5 mol) in ethyl acetoacetate (0.6 mol) at room temperature. Then the mixture was heated to 40 °C for 20–24 h. After the reaction was completed, ethanol (50 mL) was added and the mixture was stirred for another 0.5 h. The mixture was cooled to 0 °C, separated by ﬁltration, washed with cool ethanol. The crude products were purified by recrystallization from ethanol to give compounds **4a–b** (68–72%) as white crystals. Compound **4a** was prepared from 4-chloroaniline, and compound **4b** was prepared from 4-fluoroaniline.

### 3.3. General Procedure for the Preparation of 6-Substituted-2-methylquinolin-4-ols ***5a–b***

Compounds **4** (0.2 mol) was added portionwise to a stirred solution of diphenyl ether (200 mL) at 250 °C within 10 min. The resulting solution was stirred for 20 min and cooled to 40 °C, then a solid formed. Ether (150 mL) was added, the mixture was stirred for a while and cooled to room temperature. The precipitate was ﬁltered and washed with ether to afford compounds **5a–b** (92–95%) as gray solids.

### 3.4. General Procedure for the Preparation of 6-Substituted-4-chloro-2-methylquinolines ***6a–b***

A mixture of compound **5** (0.1 mol) in phosphorus oxychloride (70 mL) were heated to 80 °C for 1.5 h. Excess phosphorus oxychloride was then removed under reduced pressure. The residue was poured into ice water (500 mL), stirred intensively for 1 h and ﬁltered in order to remove insoluble solids. The filtrate was neutralized with aqueous ammonia to a pH of 8–9, ﬁltered and dried to give compounds **6a–b** (97–98%) as gray or light pink solids.

### 3.5. General Procedure for the Preparation of 6-Substituted-2-methyl-4-aminoquinolines ***7a–f***

A mixture of compounds **6a–b** (50 mmol) and aliphatic amines (0.2 mol) was stirred at reflux for 6–10 h and then cooled to room temperature. Water (200 mL) was added to the solution, and the product was precipitated and collected, washed several times with water, and dried to afford compounds **7a–f** (95–99%) as pale solids. Compound **7a** was prepared from **6a** and *N*,*N*-dimethyl-ethylenediamine. Compound **7b** was prepared from **6a** and *N*,*N*-dimethyl-1,3-propanediamine. Compound **7c** was prepared from **6a** and *N*-(3-aminopropyl)morpholine. Compound **7d** was prepared from **6b** and *N*,*N*-dimethylethylenediamine. Compound **7e** was prepared from **6b** and *N*,*N*-dimethyl-1,3-propanediamine. Compound **7f** was prepared from **6b** and *N*-(3-aminopropyl)morpholine.

### 3.6. General Procedure for the Preparation of Compounds ***8a–l***

Anhydrous zinc chloride (0.5 mmol) was added rapidly to a mixture of compound **7 **(1 mmol), aryl aldehyde (1.1 mmol) and DMF (1 mL). The reaction mixture was refluxed for 20–60 min and cooled to 70 °C, methanol (8 mL) was added and refluxed for 1 h. After cooling, the yellow solid was precipitated, ﬁltered and washed with methanol. The crude product was purified by recrystallization from methanol/DMF to afford compounds **8a–l**.

(*E)-2-(2-(Benzo[d]* [1,3]*dioxol-5-yl)vinyl)-6-chloro-N-(3-morpholin-4ylpropyl)quinolin-4-amine* (**8a**). Prepared from **7c** and heliotropin. Yield: 88%; m.p.: 189–192 °C; MS (ESI) *m/z*: 452.2, 454.1 [M+H^+^]; ^1^H-NMR (600 MHz, DMSO-*d*_6_) δ (ppm): 8.32 (s, 1H), 7.77 (d, *J* = 9.0 Hz, 1H), 7.67 (d, *J* = 16.2 Hz, 1H), 7.59 (d, *J* = 9.0 Hz, 1H), 7.36 (s, 1H), 7.33 (s, 1H), 7.18 (d, *J* = 16.2 Hz, 1H), 7.15 (d, *J* = 7.8 Hz, 1H), 6.96 (d, *J* = 7.8 Hz, 1H), 6.72 (s, 1H), 6.07 (s, 2H), 3.62 (t, 4H), 3.38 (q, 2H), 2.46 (t, 2H), 2.41 (s, 4H), 1.90–1.85 (qui, 2H).

*(E)-6-Chloro-N-(3-morpholinopropyl)-2-(2-(thiophen-2-yl)vinyl)quinolin-4-amine*(**8b**). Prepared from **7c** and thiophene-2-carbaldehyde. Yield: 79%; m.p.: 179–183 °C; MS (ESI) *m/z*: 414.1, 416.0 [M+H^+^]; ^1^H-NMR (300 MHz, DMSO-*d*_6_) δ (ppm): 8.30 (s, 1H), 7.92 (d, *J* = 15.6 Hz, 1H), 7.75 (d, *J* = 9.0 Hz, 1H), 7.57 (t, *J* = 9.0 Hz, 2H), 7.38 (d, *J* = 2.7 Hz, 1H), 7.29 (t, 1H), 7.12 (t, 1H), 7.00 (d, *J* = 15.9 Hz, 1H), 6.75 (s, 1H), 3.62 (t, 4H), 3.38(q, 2H), 2.46–2.36 (m, 6H), 1.91–1.80 (qui, 2H).

*(E)-N^1^-(6-Chloro-2-(2-(thiophen-2-yl)vinyl)quinolin-4-yl)-N^2^,N^2^-dimethylethane-1,2-diamine* (**8c**). Preparedfrom **7a** and thiophene-2-carbaldehyde. Yield: 75%; m.p.: 134–136 °C; MS (ESI) *m/z*: 358.1, 360.1 [M+H^+^]; ^1^H-NMR (600 MHz, DMSO-*d*_6_) δ (ppm): 8.29 (s, 1H), 7.92 (d, *J* = 15.0 Hz, 1H), 7.75 (d, *J* = 7.8 Hz, 1H), 7.62–7.50 (m, 2H), 7.36 (s, 1H), 7.10 (s, 1H), 7.05 (s, 1H), 7.00 (d, *J* = 15.0 Hz, 1H), 6.76 (s, 1H), 3.41 (s, 2H), 2.59 (s, 2H), 2.24 (s, 6H).

*(E)-N^1^-(6-Chloro-2-(4-(methylsulfonyl)styryl)quinolin-4-yl)-N^3^,N^3^-dimethylpropane-1,3-diamine* (**8d**). Prepared from **7b** and *p*-methylsulphonyl benzaldehyde. Yield: 87%; m.p.: 176–181 °C; MS (ESI) *m/z*: 444.3, 446.3 [M+H^+^]; ^1^H-NMR (300 MHz, DMSO-*d*_6_) δ (ppm): 8.36 (s, 1H), 7.96 (t, 4H), 7.90–7.78 (m, *J* = 16.2, 9.3 Hz, 2H), 7.62 (d, *J* = 9.0 Hz, 1H), 7.53 (d, *J* = 16.2 Hz, 1H), 7.43 (t, 1H), 6.88 (s, 1H), 3.25 (s, 3H), 3.41 (s, 2H), 2.73 (t, 2H), 2.44 (s, 6H), 2.02–1.91 (qui, 2H).

*(E)-N^1^-(6-Chloro-2-(4-methoxystyryl)quinolin-4-yl)-N^2^,N^2^-dimethylethane-1,2-diamine* (**8e**). Prepared from **7a** and *p*-anisaldehyde. Yield: 83%; m.p.: 169–172 °C; MS (ESI) *m/z*: 382.1, 384.1 [M+H^+^]; ^1^H-NMR (300 MHz, DMSO-*d*_6_) δ (ppm): 8.33 (d, *J* = 1.4 Hz, 1H), 7.74 (t, *J* = 16.2, 9.0 Hz, 2H), 7.65 (d, *J* = 8.7 Hz, 2H), 7.59 (dd, *J* = 9.0, 1.4 Hz, 1H), 7.16 (t, *J* = 16.2 Hz, 2H), 6.99 (d, *J* = 8.7 Hz, 2H), 6.79 (s, 1H), 3.80 (s, 3H), 3.48 (q, 2H), 2.71 (t, 2H), 2.34 (s, 6H).

*(E)-6-Chloro-2-(4-methoxystyryl)-N-(3-morpholin-4ylpropyl)quinolin-4-amine*(**8f**). Prepared from **7c** and *p*-anisaldehyde. Yield: 85%; m.p.: 126–129 °C; MS (ESI) *m/z*: 438.4, 440.4 [M+H^+^]; ^1^H-NMR (600 MHz DMSO-*d*_6_) δ (ppm): 8.28 (d, *J* = 1.8 Hz, 1H), 7.75 (d, *J* = 9.0 Hz, 1H), 7.67 (d, *J* = 16.2 Hz, 1H), 7.63 (d, *J* = 8.4 Hz, 2H), 7.56 (dd, *J* = 9.0, 1.8 Hz, 1H), 7.23 (t, 1H), 7.15 (d, *J* = 15.6 Hz, 1H), 6.97 (d, *J* = 8.4 Hz, 2H), 6.72 (s, 1H), 3.79 (s, 3H), 3.60 (s, 4H), 3.38-3.35 (m, 2H), 2.43 (t, 2H), 2.38 (s, 4H), 1.89–1.83 (qui, 2H).

*(E)-N^1^-(2-(2-(Benzo[d]* [1,3]*dioxol-5-yl)vinyl)-6-fluoroquinolin-4-yl)-N^2^,N^2^-dimethylethane-1,2-diamine* (**8g**). Prepared from **7d** and heliotropin. Yield: 92%; m.p.: 152–155 °C; MS (ESI) *m/z*: 380.4, 381.4 [M+H^+^]; ^1^H-NMR (300 MHz, DMSO-*d*_6_) δ (ppm): 8.04 (dd, *J* = 10.5 Hz, 1H), 7.83–7.78 (m, 1H), 7.64 (d, *J* = 16.5 Hz, 1H), 7.49 (t, 1H), 7.37 (s, 1H), 7.16 (t,*J* = 16.2, 7.2 Hz, 2H), 6.95 (d, *J* = 8.0 Hz, 2H), 6.73 (s, 1H), 6.07 (s, 2H), 3.42 (q, 2H), 2.59 (t, 2H), 2.25 (s, 6H).

*(E)-N^1^-(6-Fluoro-2-(2-(thiophen-2-yl)vinyl)quinolin-4-yl)-N^3^,N^3^-dimethylpropane-1,3-diamine* (**8h**). Prepared from **7e** and thiophene-2-carbaldehyde. Yield: 78%; m.p.: 154–157 °C; MS (ESI) *m/z*: 356.3, 357.3 [M+H^+^]; ^1^H-NMR (300 MHz, DMSO-*d*_6_) δ (ppm): 7.99 (dd, *J* = 11.1, 2.1 Hz, 1H), 7.89 (d, *J* = 15.9 Hz, 1H), 7.80 (dd, *J* = 9.0, 6.0 Hz, 1H), 7.55 (d, *J* = 5.1 Hz, 1H), 749 (td, *J* = 9.0, 8.1 Hz, 1H), 7.37 (d, *J* = 3.3 Hz, 1H), 7.19–7.10 (m, *J* = 4.2 Hz, 2H), 7.01 (d, *J* = 15.9 Hz, 1H), 6.74 (s, 1H), 2.37 (t, 2H), 2.18 (s, 6H), 1.89–1.77 (qui, 2H).

*(E)-N^1^-(6-Fluoro-2-(2-(thiophen-2-yl)vinyl)quinolin-4-yl)-N^2^,N^2^-dimethylethane-1,2-diamine* (**8i**). Prepared from **7d** and thiophene-2-carbaldehyde. Yield: 82%; m.p.: 148–152 °C; MS (ESI) *m/z*: 342.1, 343.1 [M+H^+^]; ^1^H-NMR (300 MHz, DMSO-*d*_6_) δ (ppm): 8.07 (dd, *J* = 11.7 Hz, 1H), 7.92 (d, *J* = 15.9 Hz, 1H), 7.81 (dd, *J* = 9.0, 6.0 Hz, 1H), 7.56 (d, *J* = 4.8 Hz, 1H), 7.50 (td, *J* = 9.0, 8.4 Hz, 1H), 7.37 (d, *J* = 4.5 Hz, 1H), 7.12 (t, *J* = 4.5, 3.9 Hz, 1H), 7.01 (d, *J* = 15.9 Hz, 2H), 6.77 (s, 1H), 2.63 (t, 2H), 2.28 (s, 6H).

*(E)-N^1^-(6-Fluoro-2-(4-(methylsulfonyl)styryl)quinolin-4-yl)-N^3^,N^3^-dimethylpropane-1,3-diamine* (**8j**). Prepared from **7e** and *p*-methylsulphonyl benzaldehyde. Yield: 89%; m.p.: 177–180 °C; MS (ESI) *m/z*: 428.1, 429.1 [M+H^+^]; ^1^H-NMR (300 MHz, DMSO-*d*_6_) δ (ppm): 8.05 (dd, *J* = 10.7 Hz, 1H), 8.00–7.92 (m, *J* = 8.7 Hz, 4H), 7.88–.77 (m, *J* = 16.2, 9.0, 6.0 Hz, 2H), 7.59–7.48 (m, *J* = 15.9, 9.0 Hz, 2H), 7.26 (t, 1H), 6.82 (s, 1H), 3.40 (q, 2H), 3.25 (s, 3H), 2.38 (t, 2H), 2.19 (s, 6H), 1.91–1.79 (qui, 2H).

*(E)-N^1^-(6-Fluoro-2-(4-(methylsulfonyl)styryl)quinolin-4-yl)-N^2^,N^2^-dimethylethane-1,2-diamine* (**8k**). Prepared from **7d** and *p*-methylsulphonyl benzaldehyde. Yield: 86%; m.p.: 178–182 °C; MS (ESI) *m/z*: 414.2, 416.2 [M+H^+^]; ^1^H-NMR (600 MHz, DMSO-*d*_6_) δ (ppm): 8.08 (dd, *J* = 10.8 Hz, 1H), 7.96 (q, *J* = 8.4 Hz, 4H), 7.85 (dd, *J* = 9.6, 6.0 Hz, 1H), 7.82 (d, *J* = 16.2 Hz, 1H), 7.53 (d, *J* = 15.6 Hz, 2H), 7.07 (t, 1H), 6.85 (s, 1H), 3.47–3.44 (q, 2H), 3.25 (s, 3H), 2.63 (t, 2H), 2.27 (s, 6H).

*(E)-6-Fluoro-2-(4-methoxystyryl)-N-(3-morpholin-4ylpropyl)quinolin-4-amine* (**8l**). Prepared from **7f** and *p*-anisaldehyde. Yield: 82%; m.p.: 148–151 °C; MS (ESI) *m/z*: 422.2, 423.2 [M+H^+^]; ^1^H-NMR (300 MHz, DMSO-*d*_6_) δ (ppm): 8.01 (dd, *J* = 11.1 Hz, 1H), 7.81 (dd, *J* = 9.3, 5.7 Hz, 1H), 7.70–7.61 (m,*J* = 16.5, 8.4 Hz, 3H), 7.49 (td, *J* = 9.0, 8.4 Hz, 1H), 7.16 (d, *J* = 16.2 Hz, 1H), 7.07 (t, 1H), 6.99 (d, *J* = 8.7 Hz, 2H), 6.73 (s, 1H), 3.80 (s, 3H), 3.65–3.59 (t, 4H), 3.43–3.37 (q, 2H), 2.47–2.36 (m, 6H), 1.93–1.81 (qui, 2H).

### 3.7. 4-Chloro-6-fluoro-2-methylquinoline 1-oxide (***9***)

3-Chloroperbenzoic acid (19.0 g, 0.11 mol) was added portionwise to a stirred solution of 4-chloro-6-fluoro-2-methylquinoline (**6b**, 19.6 g, 0.1 mol) in 1,2-dichloroethane (100 mL). The resulting mixture was stirred at 50 °C for 3 h and cooled to room temperature. Then the solution was washed with 0.1 mol/L NaHCO_3_ aqueous solution and brine, dried (MgSO_4_), filtered and concentrated to provide compound **9** as a yellow solid (19.7 g, 93.1%).

### 3.8. 4-Chloro-2-(chloromethyl)-6-fluoroquinoline (***10***)

Benzenesulfonyl chloride (28.2 mL, 0.22 mol) was added dropwise to a mixture of compound **9** (19.0 g, 0.09 mol) in toluene (95 mL) at 80 °C. Then the mixture was stirred for 3 h and cooled to room temperature. The solution was washed with 0.1 mol/L NaHCO_3_ aqueous solution and brine, dried (MgSO_4_), filtered and concentrated to provide a black oil. Ethanol (12 mL) was added and the mixture was stirred at 0 °C until solidified. The precipitate was filtered and washed with cold ethanol to afford compound **10** as yellow-green solid (11.6 g, 56.0%).

### 3.9. General Procedure for the Preparation of Compounds ***11a–d***

A mixture of corresponding substituted phenythiol or benzylthiol (0.055 mol) and potassium carbonate was stirred in acetonitrile at 50 °C for 10 min, then compound **10** (11.5 g, 0.05 mol) was added. The reaction mixture was stirred for 2 h, cooled to room temperature and concentrated. The residue was poured into water (150 mL), stirred for 0.5 h and separated by ﬁltration to give compounds **11a–d** as white solids (57.7–72.4%). Compound **11a** was prepared from 4-methylthiophenol, MS (ESI) *m/z*: 317.1, 318.1 [M+H^+^]. Compound **11b** was prepared from 4-methoxyphenthiol, MS (ESI) *m/z*: 333.2, 334.2 [M+H^+^]. Compound **11c** was prepared from furan-2-carbaldehyde, MS (ESI) *m/z*: 307.0, 308.0 [M+H^+^]. Compound **11d** was prepared from 4-fluorobenzylthiol, MS (ESI) *m/z*: 335.1, 336.1 [M+H^+^].

### 3.10. General Procedure for the Preparation of Compounds ***12a–d***

30% Hydrogen peroxide (28.2 mL, 0.2 mol) was added dropwise to a stirred mixture of compound **11** (0.02 mol) and sodium tungstate (2 mmol) in acetic acid (60 mL). Then the mixture was stirred at 30 °C for 6 h, poured into water (150 mL), filtered and washed with water to give compounds **12a–d** as white solids (87.2–91.5%). Compound **12a**, MS (ESI) *m/z*: 349.1, 350.0 [M+H^+^]. Compound **12b**, MS (ESI) *m/z*: 365.2, 366.2 [M+H^+^]. Compound **12c**, MS (ESI) *m/z*: 339.0, 340.0 [M+H^+^]. Compound **12d**, MS (ESI) *m/z*: 367.1, 368.1 [M+H^+^].

### 3.11. General Procedure for the Preparation of Compounds ***13a–h***

A mixture of compound **12** (2 mmol) and the corresponding aliphatic amine (8 mmol) was stirred at reflux for 2-5 h and then cooled to room temperature. Water (200 mL) was added, and the product was precipitated and collected, washed several times with water, and dried. The crude product was purified by silica gel chromatography (MeOH:CH_2_Cl_2_ = 20:1) to afford the title compounds **13a–h** (65–84%) as white solids.

*N^1^-(6-Fluoro-2-(((4-methylphenyl)sulfonyl)methyl)quinolin-4-yl)-N^2^,N^2^-dimethylethane-1,2-diamine* (**13a**). Prepared from **12a** and *N*,*N*-dimethylethylenediamine. Yield: 72%; m.p.: 158-159 °C; MS (ESI) *m/z*: 402.0, 402.9 [M+H^+^]; ^1^H-NMR (300 MHz, DMSO-*d*_6_) δ (ppm): 8.03 (d, *J* = 9.9 Hz, 1H), 7.75-7.60 (m, *J* = 9.6 7.8, 6.6 Hz, 3H), 7.49 (t, *J* = 8.4, 7.5 Hz, 1H), 7.38 (d, *J* = 7.8 Hz, 2H), 7.12 (s, 1H), 6.29 (s, 1H), 4.73 (s, 2H), 3.17 (q, 2H), 2.47 (t, 2H), 2.38 (s, 3H), 2.21 (s, 6H).

*6-Fluoro-2-(((4-methylphenyl)sulfonyl)methyl)-N-(3-morpholin-4ylpropyl)quinolin-4-amine* (**13b**). Prepared from **12a** and *N*-(3-aminopropyl)morpholine. Yield: 80%; m.p.: 183–184 °C; MS (ESI) *m/z*: 458.0, 459.9 [M+H^+^]; ^1^H-NMR (300 MHz, DMSO-*d*_6_) δ (ppm): 8.00 (d, *J* = 10.8 Hz, 1H), 7.67 (m, *J* = 10.2, 8.4, 6.6 Hz, 3H), 7.49 (t, *J* = 9.0, 8.4 Hz, 1H), 7.38 (d, *J* = 7.5 Hz, 2H), 7.17 (s, 1H), 6.31 (s, 1H), 4.71 (s, 2H), 3.60 (t, 4H), 3.15 (q, 2H), 2.37 (d, 9H), 1.79–1.70 (qui, 2H).

*6-Fluoro-2-(((4-methoxyphenyl)sulfonyl)methyl)-N-(3-morpholin-4ylpropyl)quinolin-4-amine* (**13c**). Prepared from **12b** and *N*-(3-aminopropyl)morpholine. Yield: 84%; m.p.: 174–177 °C; MS (ESI) *m/z*: 474.0 [M+H^+^]; ^1^H-NMR (300 MHz, DMSO-*d*_6_) δ (ppm): 8.01 (dd, *J* = 11.1, 2.4 Hz, 1H), 7.71–7.65 (m, *J* = 10.5, 8.7, 5.7 Hz, 3H), 7.49 (td, *J* = 8.7, 8.4, 2.6 Hz, 1H), 7.19 (t, 1H), 7.09 (d, *J* = 9.0 Hz, 2H), 6.30 (s, 1H), 4.69 (s, 2H), 3.83 (s, 3H), 3.60 (t, 4H), 3.15 (q, 2H), 2.37 (t, 6H), 1.80–1.71 (qui, 2H).

*N^1^*-(6-fluoro-2-(((furan-2-ylmethyl)sulfonyl)methyl)quinolin-4-yl)-*N^2^,N^2^*-dimethylethane-1,2-diamine (**13d**). Prepared from **12c** and *N*,*N*-dimethylethylenediamine. Yield: 65%; m.p.: 147–149 °C; MS (ESI) *m/z*: 392.1, 393.1 [M+H^+^]; ^1^H-NMR (300 MHz, DMSO-*d*_6_) δ (ppm): 8.05 (d, *J* = 10.8 Hz, 1H), 7.87 (dd, *J* = 9.3, 6.0 Hz, 1H), 7.73 (d, 1H), 7.56 (td, *J* = 8.4, 6.3 Hz, 1H), 7.17 (t, 1H), 6.64 (d, *J* = 2.7 Hz, 2H), 6.52 (t, 1H), 4.80 (s, 2H), 4.61 (s, 2H), 3.38 (q, 2H), 2.56 (t, 2H), 2.23 (s, 6H).

*6-Fluoro-2-(((furan-2-ylmethyl)sulfonyl)methyl)-N-(3-morpholin-4ylpropyl)quinolin-4-amine* (**13e**). Prepared from **12c** and *N*-(3-aminopropyl)morpholine. Yield: 76%; m.p.: 155–157 °C; MS (ESI) *m/z*: 448.0, 449.0 [M+H^+^]; ^1^H-NMR (300 MHz, DMSO-*d*_6_) δ (ppm): 8.06 (dd, *J* = 10.8, 2.5 Hz, 1H), 7.87 (dd, *J* = 9.3, 6.0 Hz, 1H), 7.74 (s, 1H), 7.56 (td, *J* = 9.0, 8.4, 2.5 Hz, 1H), 7.29 (t, 1H), 6.65 (d, *J* = 3.0 Hz, 1H), 6.62 (s, 1H), 6.53 (t, 1H), 4.81 (s, 2H), 4.61 (s, 2H), 3.58 (t, 4H), 3.33 (s, 2H), 2.39 (t, 6H), 1.86–1.81 (qui, 2H).

*N^1^-(6-fluoro-2-(((4-fluorobenzyl)sulfonyl)methyl)quinolin-4-yl)-N^2^,N^2^-dimethylethane-1,2-diamine* (**13f**). Prepared from **12d** and *N*,*N*-dimethylethylenediamine. Yield: 72%; m.p.: 177–180 °C; MS (ESI) *m/z*: 420.0, 421.0 [M+H^+^]; ^1^H-NMR (300 MHz, DMSO-*d*_6_) δ (ppm): 8.07 (d, *J* = 10.8 Hz, 1H), 7.92 (dd, *J* = 8.7, 5.7 Hz, 1H), 7.60 (d, 3H), 7.26 (t, *J* = 8.7, 8.4 Hz, 2H), 7.18 (s, 1H), 6.65 (s, 1H), 4.65 (s, 2H), 4.52 (s, 2H), 3.39 (s, 2H), 2.58 (t, 2H), 2.24 (s, 6H).

*N^1^-(6-fluoro-2-(((4-fluorobenzyl)sulfonyl)methyl)quinolin-4-yl)-N^3^,N^3^-dimethylpropane-1,3-diamine* (**13g**). Prepared from **12d** and *N*,*N*-dimethyl-1,3-propane diamine. Yield: 77%; m.p.: 156–158 °C; MS (ESI) *m/z*: 434.0, 435.0 [M+H^+^]; ^1^H-NMR (300 MHz, DMSO-*d*_6_) δ (ppm): 8.05 (d, *J* = 10.2 Hz, 1H), 7.91 (dd, *J* = 9.0, 6.0 Hz, 1H), 7.58 (t, 3H), 7.33 (t, 1H), 7.26 (t, *J* = 8.1 Hz, 2H), 6.61 (s, 1H), 4.65 (s, 2H), 4.51 (s, 2H), 3.29 (d, 2H), 2.35 (t, 2H), 2.17 (s, 6H), 1.86–1.78 (qui, 2H).

*6-Fluoro-2-(((4-fluorobenzyl)sulfonyl)methyl)-N-(3-morpholin-4ylpropyl)quinolin-4-amine* (**13h**). Prepared from **12d** and *N*-(3-aminopropyl)morpholine. Yield: 81%; m.p.: 192–195 °C; MS (ESI) *m/z*: 476.0 [M+H^+^]; ^1^H-NMR (300 MHz, DMSO-*d*_6_) δ (ppm): 8.07 (d, *J* = 10.5 Hz, 1H), 7.91 (dd, *J* = 8.7, 6.0 Hz, 1H), 7.58 (s, 3H), 7.26 (t, *J* = 8.7 Hz, 3H), 6.62 (s, 1H), 4.65 (s, 2H), 4.51 (s, 2H), 3.58 (s, 4H), 3.31 (s, 1H), 2.38 (s, 6H), 1.86–1.81 (qui, 2H).

### 3.12. Evaluation of the Biological Activity

The antiproliferative activity of compounds **8a–l** and **13a–h** was evaluated with non-small-cell lung cancer cell line (H-460), human colorectal cancer cell line (HT-29), human liver cancer cell line (HepG2) and stomach cancer cell line (SGC-7901) by the MTT method *in vitro*, with compound Iressa as positive control. The cancer cells were cultured in minimum essential medium (MEM) supplemented with 10% fetal bovine serum (FBS). Approximately 4 × 10^3^ cells, suspended in MEM medium, were plated onto each well of a 96-well plate and incubated in 5% CO_2_ at 37 °C for 24 h. The test compounds were added to the culture medium at the indicated final concentrations and the cell cultures were continued for 72 h. Fresh MTT was added to each well at a final concentration of 5 μg/mL and incubated with cells at 37 °C for 4 h. The formazan crystals were dissolved in 100 μL DMSO per each well, and the absorbency at 492 nm (for the absorbance of MTT formazan) and 630 nm (for the reference wavelength) was measured with the ELISA reader. All of the compounds were tested twice in each of the cell lines. The results expressed as IC_50_ (inhibitory concentration of 50%) were the averages of two determinations and were calculated by using the Bacus Laboratories Incorporated Slide Scanner (Bliss) software.

## 4. Conclusions

In conclusion, an efficient optimized method was employed to generate 2-arylvinylquinolines, using anhydrous zinc chloride in reﬂuxing DMF. Two series of novel 2-substituted-4-amino-6-halogenquinolines were synthesized and evaluated for their antiproliferative activity against four human cancer cell lines (H-460, HT-29, HepG2 and SGC-7901). The preliminary SARs showed that the improved activity depended strongly on the introduction of an ethylene linkage between the nucleus and aryl moiety. Among the tested compounds, compound **8e** with a 4-methoxystyryl group at the C-2 position and a dimethylaminoalkylamino substituent at the C-4 position is considered a promising lead for further structural modifications.
